# Comparison of Carboplatin With 5-Fluorouracil vs. Cisplatin as Concomitant Chemoradiotherapy for Locally Advanced Head and Neck Squamous Cell Carcinoma

**DOI:** 10.3389/fonc.2020.00761

**Published:** 2020-06-05

**Authors:** Saskia H. Hanemaaijer, Iris C. Kok, Rudolf S. N. Fehrmann, Bert van der Vegt, Jourik A. Gietema, Boudewijn E. C. Plaat, Marcel A. T. M. van Vugt, Marije R. Vergeer, C. René Leemans, Johannes A. Langendijk, Jens Voortman, Jan Buter, Sjoukje F. Oosting

**Affiliations:** ^1^Department of Otorhinolaryngology, University of Groningen, University Medical Center Groningen, Groningen, Netherlands; ^2^Department of Medical Oncology, University of Groningen, University Medical Center Groningen, Groningen, Netherlands; ^3^Department of Pathology, University of Groningen, University Medical Center Groningen, Groningen, Netherlands; ^4^Department of Radiation Oncology, Amsterdam UMC, Vrije Universiteit Amsterdam, Amsterdam, Netherlands; ^5^Department of Otolaryngology/Head and Neck Surgery, Amsterdam UMC, Vrije Universiteit Amsterdam, Amsterdam, Netherlands; ^6^Department of Radiotherapy, University of Groningen University Medical Center Groningen, Groningen, Netherlands; ^7^Department of Medical Oncology, Amsterdam UMC, Vrije Universiteit Amsterdam, Amsterdam, Netherlands

**Keywords:** area under the concentration–time curve, carboplatin 5-flourouracil, chemoradiotherapy, cisplatin, comparison, head and neck squamous cell carcinoma, locally advanced head and neck squamous cell carcinoma

## Abstract

**Background:** Chemoradiotherapy (CRT) including three cycles of cisplatin is considered the standard of care for locally advanced head and neck squamous cell carcinoma (LA-HNSCC). However, around one-third of the patients cannot complete cisplatin because of toxicity. Carboplatin plus 5-fluorouracil (carbo-5FU) is another accepted treatment option with a different toxicity profile. We compared tolerability and efficacy of concomitant carbo-5FU and cisplatin.

**Patients and Methods:** We conducted a retrospective analysis of LA-HNSCC patients treated with CRT in two Dutch cancer centers between 2007 and 2016. All patients received intensity-modulated radiotherapy. One center routinely administered carboplatin 300–350 mg/m^2^ at day 1, 22, and 43 followed by 5FU 600 mg/m^2^/day for 96 h. The other center used cisplatin 100 mg/m^2^ at day 1, 22, and 43. The primary endpoint of this study was chemotherapy completion rate. Secondary endpoints included overall survival (OS), disease-free survival (DFS), locoregional control (LRC) and distant metastasis–free interval (DMFS), toxicity, and unplanned admissions.

**Results:** In the carbo-5FU cohort (*n* = 211), 60.2% of the patients completed chemotherapy vs. 76.7% (*p* < 0.001) of the patients in the cisplatin cohort (*n* = 223). Univariate analysis showed a higher risk of death in the carbo-5FU cohort [hazard ratio (HR) 1.53, 95% CI, 1.09–2.14, *p* = 0.01] with a 3-year OS of 65.4 vs. 76.5% for cisplatin. OS was independently associated with T and N stage and p16 status, but not with chemotherapy regimen (HR 1.08, 95% CI, 0.76–1.55, *p* = 0.65). Three-year DFS was 70.0% for carbo-5FU vs. 78.6% for cisplatin (HR 1.37, 95% CI, 0.93–2.01, *p* = 0.05). A similar outcome was observed for both LRC (HR 1.27, 95% CI, 0.74–2.09, *p* = 0.4) and DMFS (HR 1.08, 95% CI 0.62–1.90, *p* = 0.77). The risk of discontinuation for chemotherapy-associated toxicity was higher in the carbo-5FU cohort than in the cisplatin cohort (relative risk = 1.69).

**Conclusion:** LA-HNSCC patients treated with concomitant carbo-5FU completed chemotherapy less frequently than patients treated with cisplatin. Treatment regimen was not an independent prognostic factor for OS.

## Introduction

Head and neck squamous cell carcinoma (HNSCC) is the seventh most common type of cancer worldwide, accounting for 600,000 new cases and 250,000 cancer deaths each year (1, 2). About half of the patients are diagnosed with locally advanced head and neck squamous cell carcinoma (LA-HNSCC) (3). Definitive chemoradiotherapy (CRT) is the treatment of choice in patients with unresectable LA-HNSCC. Concomitant CRT has been shown to be superior to radiotherapy alone, with an absolute survival benefit of 6.5% at 5 years, raising 5-year overall survival (OS) from 27.2 to 33.7% (4). In addition, recent studies have indicated that higher cumulative cisplatin dose is associated with better OS (5, 6).

Concomitant CRT consisting of three cycles of high-dose cisplatin on day 1, day 22, and day 43 in combination with 70 Gray (Gy) radiotherapy in 35 fractions is considered the standard of care. However, this regimen causes significant acute and late toxicity. As a result, approximately one-third of the patients cannot complete three cisplatin cycles (7). Concomitant carboplatin plus 5-fluorouracil (carbo-5FU) has also been shown to improve OS compared to radiotherapy alone and is an accepted treatment regimen (4). However, no randomized controlled trials comparing concomitant cisplatin with concomitant carbo-5FU have been conducted. To study differences in tolerability and efficacy of both regimens, we compared all consecutive patients from one Dutch cancer center that routinely used carbo-5FU with all consecutive patients from another Dutch cancer center that routinely used high-dose cisplatin.

We hypothesized that carbo-5FU is better tolerated than cisplatin and will be reflected by a higher percentage of patients completing three chemotherapy cycles, with similar efficacy. Therefore, the primary endpoint of this study was chemotherapy completion rate. Secondary endpoints included OS, disease-free survival (DFS), locoregional control (LRC) and distant metastasis–free interval, toxicity, and the number of unplanned admissions.

## Patients and Methods

### Patients, Study Design, and Data Collection

We conducted a comparative retrospective cohort study of patients who received primary concomitant CRT for LA-HNSCC at the University Medical Center Groningen (UMCG) or Amsterdam University Medical Center (AUMC) between July 2007 and February 2016. Inclusion criteria were histologically proven HNSCC, stage III–IVB and concomitant CRT as primary treatment. Patients with nasopharyngeal carcinoma and patients who received postoperative CRT or CRT for cancer recurrence were excluded. Chemotherapy other than carbo-5FU (UMCG) or 3-weekly cisplatin (AUMC) was an additional exclusion criterion. Both centers prospectively included all patients treated with CRT in a database; these databases were used to identify candidates for both cohorts. Information about patient demographics, comorbidity, smoking, tumor characteristics, treatment details, toxicity, and outcome were extracted from electronic patient records. Survival status of patients lost to follow-up was checked in the population register. The Dutch Medical Research Involving Human Subjects Act does not apply to data collection as part of routine clinical practice. Therefore, the local ethics committee determined that this study was exempt from the ethical approval requirement. The study was registered at ClinicalTrials.gov (Identifier: NCT02778191).

### Endpoints

The primary endpoint of the study was the percentage of patients completing three chemotherapy cycles, regardless of dose reduction. Secondary endpoints included overall OS, DFS, LRC and distant metastasis–free interval, toxicity, and the number of unplanned admissions. OS was defined as the time between diagnosis and death from any cause. Patients who were alive at the last follow-up were censored. DFS was defined as the time between the last day of CRT and first evidence of relapse or death from any cause, whichever occurred first. LRC was defined as the time between the last day of CRT and first evidence of local or regional relapse. Distant metastases–free interval was defined as the time between the last day of CRT and first evidence of distant metastasis. For LRC and distant metastasis–free interval, patients who died without evidence of recurrence were censored at the date of death. Toxicity was reported according to CTCAE version 4.03. Reasons for chemotherapy discontinuation were noted.

### Treatment Regimens

The carbo-5FU regimen consisted of carboplatin 300 mg/m^2^ for patients with a creatinine clearance of 80–120 mL/min and 350 mg/m^2^ for patients with a creatinine clearance of >120 mL/min. Creatinine clearance was calculated using the Cockcroft–Gault formula. Carboplatin was given intravenously in 250 mL of dextrose 5% over 30 min. 5FU was given as a continuous infusion at a dose of 600 mg/m^2^/day in 2,000 mL of normal saline with 20 mmol potassium for 96 h. The whole chemotherapy cycle was given as an inpatient regimen. Chemotherapy cycles were started on day 1, day 22, and day 43 of radiotherapy. Cisplatin 100 mg/m^2^ was given intravenously in 500 mL of normal saline over 3 h on day 1, day 22, and day 43 of RT. Prehydration consisted of 1 L of normal saline plus 20 mmol potassium and 2 g of magnesium sulfate over 2 h and posthydration consisted of 4 L of normal saline plus 80 mmol of potassium and 8 g of magnesium sulfate over 20 h. Dexamethasone and a 5-HT3 receptor antagonist were given as antiemetics with both treatment regimens; and patients treated with cisplatin also received aprepitant. All patients received intensity modulated radiotherapy with 70 Gy in 35 daily fractions of 2 Gy on weekdays on the primary tumor and involved lymph nodes. Elective areas were treated with 54.25 Gy in 35 daily fractions of 1.55 Gy.

### Clinical Evaluation and Follow-Up

During treatment, patients were evaluated weekly by a radiation oncologist for locoregional toxicity. In addition, patients were evaluated by a medical oncologist before and after every chemotherapy cycle for systemic toxicity until resolution of acute side effects. Response evaluation, including clinical examination and computerized tomography (CT) or magnetic resonance imaging (MRI), was performed 8–12 weeks after CRT. In case of residual disease, salvage surgery was performed if possible. During follow-up, patients were seen by a radiation oncologist and head-and-neck surgeon every 3 months until 2 years after CRT, and thereafter every 6 months until 5 years after CRT. Imaging using CT or MRI was done in case of clinical suspicion of recurrent disease.

### Carboplatin Area Under the Concentration–Time Curve

For patients who received carboplatin, the dose was calculated retrospectively as area under the curve (AUC) for each cycle. The Calvert formula was used: AUC = absolute dose (mg) carboplatin/(creatinine clearance + 25).

### Statistical Analysis

Assuming a completion rate of 60% for cisplatin and a sampling ratio of 1, we estimated that 353 patients had to be included to demonstrate 10% difference in completion rate in favor of carbo-5FU, with a type 1 error rate (alpha) of 5% and a power of 0.80.

Pearson chi-square test and Mann–Whitney *U*-test were used to compare variables between patient cohorts. Logistic regression analysis was used to analyze the association between chemotherapy completion and other variables. In the univariate analyses all patient and tumor characteristics were included. For the multivariate analyses only the factors that were significant in univariate analysis were used.

In the univariate analysis, OS, DFS, LRC, and distant metastasis–free interval were analyzed using the Kaplan–Meier method and compared with the log-rank test. Hazard ratios (HRs) were calculated with the Cox proportional hazards model. In both Kaplan Meier and Cox regression analysis, censoring was applied in case patients were lost to follow-up. In the multivariable analysis, associations between clinical and pathological parameters and OS were determined with Cox proportional hazards model using the Wald statistic. In addition, Cox regression provided an estimate of the effect of treatment on OS and DFS after the adjustment for covariates modeling the HR. In this way, we were able to investigate OS and DFS stratified for chemotherapy regimen independently of the differences in baseline characteristics.

Levene's test was used to compare variances in AUC. To determine if high carboplatin AUC during cycle one and two resulted in omission of the third cycle, we used the Mann–Whitney *U* test to compare the cumulative AUC dose of cycle one plus cycle two between patients who completed three carbo-FU cycles and patients who completed only two cycles. Statistical tests were performed using SPSS (version 23.0 for Windows® IBM, Armonk, NY, USA).

## Results

### Demographics

A total of 434 patients were included, 211 of whom were in the carbo-5FU cohort and 223 in the cisplatin cohort. From all consecutive patients treated at the UMCG, 30 patients received cetuximab and were therefore excluded from analysis. None of the UMCG patients was treated with cisplatin. A total of 72 patients were excluded from the AUMC cohort based on either receiving weekly cisplatin (*n* = 48) or cetuximab (*n* = 27) (Supplementary Figure 1). Of the total group of patients, 74% were men. The mean age at diagnosis was 57 years (range 29–73). The most common tumor location was the oropharynx (64%). Patient and tumor characteristics are listed in Table 1. Detailed information on comorbidity in both study cohorts is shown in Table 2. No significant differences between patients treated with carbo-5FU and cisplatin were noted for sex, age, comorbidity, tumor site, and stage. However, there were significant differences between both study cohorts for the following variables: T and N classification, p16 status, and tobacco exposure. Patients treated with carbo-5FU had higher T and N classification and fewer p16-positive oropharynx tumors and were more frequently current smokers. Because no reliable data on World Health Organization (WHO) performance score, mucositis, skin toxicity, and alcohol consumption could be derived from patient records; these variables could not be included in the analysis.

**Table 1:** Baseline patient characteristics.

	**Carbo-5FU**	**Cisplatin**	***p***
**Characteristics**	**(*n* = 211)**	**(*n* = 233)**	
**Age, years**			0.605
[Median (IQR)]	[58 (53–63)]	[58 (52–63)]	
**Sex**			0.186
Male	149 (70.6)	170 (76.2)	
Female	62 (29.4)	53 (23.8)	
**Comorbidity**			0.902
Yes	75 (35.3)	78 (35.0)	
No	136 (64.5)	145 (65.0)	
**Tumor site**			0.869
Oral cavity	14 (6.6)	25 (11.2)	
Hypopharynx	30 (14.2)	38 (17.0)	
Larynx	24 (11.4)	26 (11.7)	
Oropharynx	143 (67.8)	134 (60.1)	
**T classification**			0.002
T1	18 (8.5)	23 (10.3)	
T2	39 (18.5)	49 (22.0)	
T3	55 (26.1)	88 (39.5)	
T4	99 (46.9)	63 (28.3)	
**N classification**			<0.001
N0	14 (6.6)	40 (17.9)	
N1	17 (8.1)	42 (18.8)	
N2	171 (81.0)	135 (60.5)	
N3	9 (4.3)	6 (2.7)	
**AJCC stage**			0.650
III	27 (12.8)	45 (20.2)	
Iva	162 (76.8)	158 (70.9)	
IVb	22 (10.4)	20 (9.0)	
**p16 (in oropharynx tumors)**			<0.001
Positive	65 (45.4)	85 (63.4)	
Negative	73 (51.0)	39 (29.1)	
Unknown	5 (3.5)	10 (7.5)	
**Tobacco exposure**			0.565
Current smoker	138 (65.4)	97 (43.5)	
Former smoker	53 (25.1)	82 (36.8)	
Never smoked	20 (9.5)	44 (19.7)	
**Pack-years**			0.262
Zero	20 (9.5)	44 (19.7)	
<10	3 (1.4)	8 (3.6)	
>10	167 (79.1)	143 (64.1)	
Missing	21 (10.0)	28 (12.6)	

*Data are presented as No. (%) unless otherwise indicated. The Mann–Whitney U-test was used. AJCC, American Joint Committee on Cancer (7th edition); IQR, interquartile range. Tobacco exposure is defined as current/former smoker*.

**Table 2:** Baseline comorbidity in the carbo-5FU and in the cisplatin cohort.

	**Carbo-5FU**	**Cisplatin**	***p^a^***
**Characteristics**	**(*n* = 211)**	**(*n* = 233)**	
Comorbidity			0.902^b^
Myocardial infarction	18	19	1.00
Peripheral vascular disease	14	4	0.15
Diabetes mellitus	11	16	0.432
Pulmonary embolism	2	2	1.00
COPD	11	9	0.217
Cerebrovasculair accident/TIA	11	9	0.649
Other malignancy^c^	17	30	0.089

*COPD, chronic obstructive pulmonary disease; TIA, transient ischemic attack*.

a *Pearson chi square test was used unless otherwise indicated*.

b *Mann–Whitney U-test was used*.

c *Malignancy in medical history*.

### Treatment Compliance

Of the patients treated with carbo-5FU, 60.2% completed three chemotherapy cycles vs. 76.7% of the patients treated with cisplatin (*p* < 0.001). Only 4.4% of the patients in the carbo-5FU cohort and 6.7% of the patients in the cisplatin cohort had chemotherapy dose reductions (*p* = 0.3). The reasons for discontinuation of chemotherapy are listed in Table 3.

**Table 3:** Reasons for discontinuation of chemotherapy.

	**Carbo-5FU**	**Cisplatin**	***p***
**Reasons^a^**	**(*n* = 211)**	**(*n* = 233)**	
Thrombocytopenia	52 (24.6)	1 (0.4)	<0.001
Acute kidney injury	2 (0.9)	21 (9.4)	<0.001
Leukocytopenia	19 (9.0)	6 (2.6)	0.005
Ototoxicity	0 (0)	8 (3.5)	0.005
Emesis	0 (0)	5 (2.2)	0.029
Performance	4 (1.9)	1 (0.4)	0.158
Patient request	5 (2.3)	2 (0.9)	0.223
Allergic reaction	2 (0.9)	0 (0)	0.145
Anemia	4 (1.9)	1 (0.4)	0.158
Other	11 (1.4)	8 (3.1)	0.408

Data presented as No. (%) unless otherwise indicated. The Mann–Whitney U-test was used.

a *One or more reasons could be reported per patient*.

### Outcome

The median follow-up was 27 months (range 1–109) in the carbo-5FU cohort and 35 months (range 1–111) in the cisplatin cohort. In the carbo-5FU cohort, 79 patients had died (37.4%), compared to 61 (27.4%) in the cisplatin cohort (Table 4). The cause of death was tumor-related in 53 patients (25.1%) in the carbo-5FU cohort and in 45 patients (20.2%) in the cisplatin cohort. Treatment-related deaths were observed in 3 patients (1.4%) in the carbo-5FU cohort (heart failure, sepsis, and sudden death) and in 2 patients (0.9%) in the cisplatin cohort (pneumonia and sudden death).

**Table 4:** Disease and survival status of patients at the time of analysis.

	**Carbo-5FU**	**Cisplatin**	***p*^**a**^**
	***n* = 211**	***n* = 233**	
**Survival status**			0.03
NED	128 (60.7)	155 (69.5)	
AWD	4 (1.9)	7 (3.1)	
DOD	52 (24.6)	44 (19.7)	
DOC	26 (12.3)	17 (7.6)	
Missing death	1 (0.5)	0 (0)	
**Event**			
Residual disease	13 (6.2)	5 (2.2)	0.041
Local regional recurrence	28 (13.3)	30 (13.5)	0.611
Distant metastasis	24 (11.3)	25 (11.2)	0.957
Local regional + distant metastasis	49 (23.2)	47 (21.1)	0.590

a * Mann–Whitney U-test was used. NED, no evidence of disease; AWD, alive with disease; DOD, died of disease; DOC, died of other cause*.

**Figure 1:**
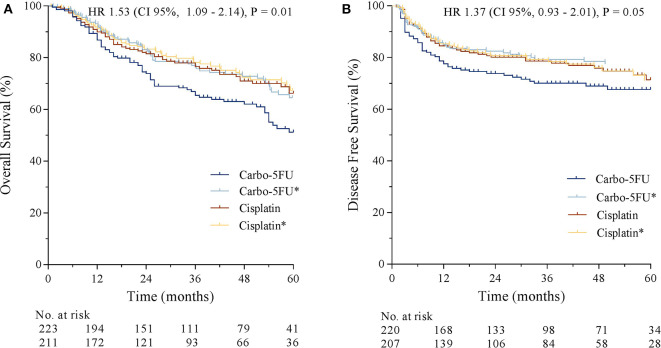
**(A)** Overall survival stratified by chemotherapy regimen. **(B)** Disease-free survival stratified by chemotherapy regimen. The adjusted overall survival estimation for both cohorts is labeled with an asterisk.

Median OS was 65 months in the carbo-5FU cohort and not reached in the cisplatin cohort. The risk of death was higher in the carbo-5FU cohort (HR 1.53, 95% CI, 1.09–2.14, *p* = 0.01). 1-year and 3-year OS was 86.7 and 65.5% for carbo-5FU and 89.9% and 76.6% for cisplatin (Figure 1A). Univariate analysis demonstrated that lower T-classification, lower N-classification, p16 positivity (in oropharyngeal tumors), non-smoking, cisplatin chemotherapy, and the absence of a second primary tumor were associated with better OS (Table 5). Sex, age, and tumor site were not associated with OS. Multivariate analysis showed that lower T classification and lower N classification, p16 positivity (in oropharyngeal tumors), and the absence of second primaries were independently associated with better OS (Table 5). Chemotherapy regimen (HR 1.08, 95% CI, 0.76–1.55) and smoking (HR 1.20, 95% CI, 0.91–1.60) were not independently associated with OS. Kaplan–Meier curves for OS of both study cohorts and adjusted OS curves according to the Cox proportional hazards model (labeled with an asterisk) are shown in Figure 1A.

**Table 5:** Significant Variables Univariate Analysis and Multivariate Analysis for Overall Survival.

**Variable**	**HR**	**95% CI**	***p***
**Univariate analysis**			
T-classification T1-T2	1(ref)		
T3-T4	4.04	2.400–6.803	<0.001
N-classification N0-N2a	1(ref)		
N2bc-N3	1.83	1.244–2.700	0.002
p16 (in oropharynx tumors)	0.25	0.153–0.411	<0.001
Second primary^a^	2.38	1.252–4.541	0.008
Treatment regimen	1.53	1.094–2.136	0.013
Tobacco exposure	1.30	1.779–1.669	0.041
**Multivariate analysis**			
T-classification T1-T2	1(ref)		
T3-T4	3.42	1.984–5.882	<0.001
N-classification N0-N2a	1(ref)		
N2bc-N3	2.23	1.464–3.380	<0.001
p16 (in oropharynx tumors)	0.35	0.211–0.591	<0.001
Second primary^a^	2.98	1.464–5.851	0.001
Treatment regimen	1.19	0.835–1.709	0.331
Tobacco exposure	1.15	0.859–1.538	0.349

*Cox regression was used. (ref) denotes for reference variable. Tobacco exposure was defined as current/former smoker or non-smoker*.

a *Second primary in the Head and Neck region at time of initial diagnosis*.

**Figure 2:**
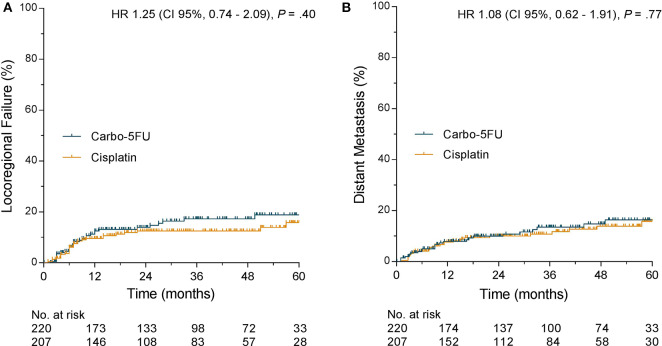
**(A)** Locoregional failure stratified by chemotherapy regimen. **(B)** Distant metastasis-free interval stratified by chemotherapy regimen.

Median DFS was not reached in either cohort. One-year and 3-year DFS were 77.5 and 70.0% for carbo-5FU compared to 84.5 and 78.6% for cisplatin (HR 1.37, 95% CI, 0.93– 2.01, *p* = 0.05, Figure 1B). Kaplan–Meier curves for DFS of both study cohorts and adjusted DFS curves according to the Cox proportional hazard model (labeled with an asterisk) are shown in Figure 1B. Similar outcome was observed for both LRC (HR 1.27, 95% CI, 0.74– 2.09, *p* = 0.4) and distant metastasis-free interval (HR 1.08, 95% CI, 0.62–1.90, *p* = 0.77) as presented in Figure 2.

**Figure 3:**
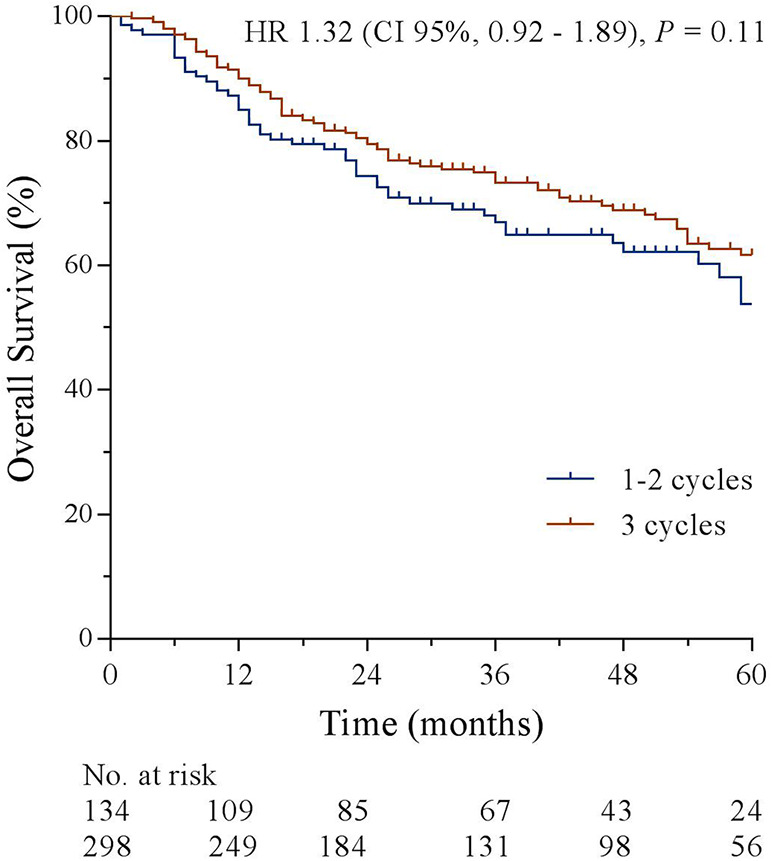
Overall survival stratified by chemotherapy completion.

In a combined analysis of the treatment groups, a trend toward better OS in patients who completed three cycles of chemotherapy compared to the patients who completed one or two cycles of chemotherapy was observed (5-year OS 61.6 vs. 53.7%, HR 1.32, 95% CI, 0.92–1.89, *p* = 0.11); see Figure 3.

### Treatment-Related Toxicity

Thrombocytopenia was the main reason for discontinuation in the carbo-5FU cohort. Acute kidney injury followed by ototoxicity was the most common reason for not completing three cycles of cisplatin. The risk of discontinuation for chemotherapy-associated toxicity was higher in the carbo-5FU cohort than in the cisplatin cohort (relative risk = 1.69). There was no difference in radiotherapy completion rate between study cohorts: 97.7% for the carbo-5FU cohort vs. 95.5% for the cisplatin cohort (*p* = 0.3). In multivariate analysis, chemotherapy completion was negatively associated with carbo-5FU, female sex, and comorbidity. No association between chemotherapy completion rate and T classification, N classification, tumor location, age, smoking status, or p16 status was found.

Grade 3 or 4 anemia was seen only in the carbo-5FU cohort and occurred in 4.7% of the patients. Grade 3 or 4 thrombocytopenia was observed more frequently in the carbo-5FU cohort compared to the cisplatin cohort (13.7 vs. 1.8%, *p* < 0.001). Neutropenic fever was uncommon in both cohorts, whereas leukopenia was frequently reported in both groups: in 15.6% of the patients in the carbo-5FU cohort and in 21.1% in the cisplatin cohort (*p* = 0.430). Acute kidney injury grade 3–4 was observed only in the cisplatin cohort, in 13 patients (6 vs. 0%, *p* < 0.001). At 6–12 weeks after treatment, 7 of these patients (3%) still had treatment-related kidney injury.

**Figure 4:**
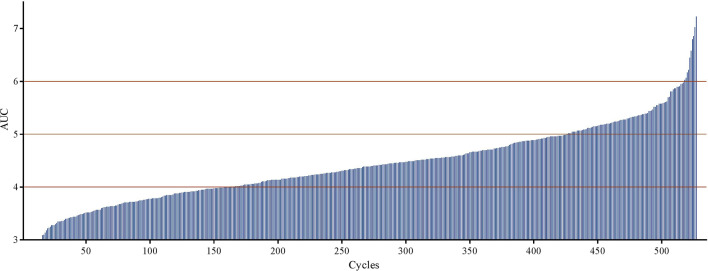
Distribution of carboplatin AUC. Each vertical bar on the horizontal axis represents a chemotherapy cycle.

Almost 80% of the patients in the carbo-5FU cohort received carboplatin, with an AUC between 4 and 6 (Figure 4). The mean AUC of the cycles received was <4.00 in 36 patients (17%) and >7.00 in 8 patients (3.8%). Four of these 8 patients could not continue therapy because of treatment-related toxicity. Patients who completed three carbo-5FU cycles received a similar cumulative carboplatin dose during the first two cycles [median 8.78, interquartile range (IQR) 7.90–9.75, min–max 3.28–12.40] compared to patients that completed two carbo-5FU cycles (median 8.71, IQR 7.84–9.74, min–max 4.52–13.83, *p* = 0.85). No association was found between carboplatin dose and grade 3 or 4 thrombocytopenia, leukocytopenia, or anemia.

### Extra Hospital Admissions

Patients treated with cisplatin were more likely to have extra hospital admissions (57.4 vs. 18.5%, *p* < 0.001) during CRT, mainly due to dehydration/acute kidney injury. The median duration of unplanned admissions was 4 days in the cisplatin cohort, with an IQR of 4–9 (range 1–28) and 5 days in the carbo-5FU cohort (IQR 3–7, range 1–60). Reasons for each extra hospital admission are reported in Table 6.

**Table 6:** Reasons Extra Hospital Admission.

	**Carbo-5FU**	**Cisplatin**	***p*^a^**
	***n* = 211**	***n* = 233**	
**Characteristics**			
Extra admission			>0.001
One	39 (18.5)	128 (57.4)	
Two	5 (2.4)	53 (23.8)	
Duration of admission			0.171
[median(IQR)]	[5(4–9)]	[4(3–7)]	
Reason for admission			>0.001
Decreased renal function	0 (0)	79 (35.4)	
Nausea and vomiting	6 (2.8)	44 (19.7)	
Fever	22 (10.4)	14 (6.27)	
Dehydration	1 (0.5)	15 (6.7)	
Diminished performance	3 (1.4)	8 (3.1)	
Infection	3 (1.4)	7 (2.7)	
Tumor related	3 (1.4)	1 (0.4)	
Other	6 (2.8)	13 (5.8)	

a *Mann-Whitney U test was used. IQR, inter quartile range*.

## Discussion

The purpose of this study was to compare tolerability and efficacy of concomitant CRT with carbo-5FU and concomitant CRT with cisplatin in locally advanced HNSCC. We hypothesized that carbo-5FU is better tolerated than cisplatin and therefore results in a higher percentage of patients completing three chemotherapy cycles with similar efficacy. However, the results showed that patients in the carbo-5FU cohort were less likely to complete three cycles of chemotherapy than patients in the cisplatin cohort.

The observed completion rate of 76.8% in the cisplatin cohort was higher than expected, and even higher than in two randomized controlled trials, where one would expect selection of fitter patients compared to our real-life data (7, 8). A factor that might have influenced cisplatin completion rate is the proportion of p16-positive oropharyngeal cancer patients, which is rising over time. Since p16-positive oropharyngeal cancer usually occurs in younger patients with a better performance status, fewer smoking pack-years, and less comorbidity, it is likely that these patients are more often capable of completing three cycles. However, we found no association between p16 status and chemotherapy completion. Another factor could be the rigorous supportive care given by specialized oncology nurse practitioners. Vigorous hydration and frequent extra admissions could possibly have led to higher chemotherapy compliance and therefore should be taken into consideration for generalizability of the results.

In a trial comparing CRT with carbo-5FU to RT alone for stage III–IVB HNSCC, 65% of the patients completed three cycles of chemotherapy (9). This is comparable to the completion rate (60.2%) that we found. In our study, the reasons for not completing chemotherapy differed between cohorts, with more thrombocytopenia and leucopenia reported in the carbo-5FU cohort and more kidney injury and ototoxicity in the cisplatin cohort. This corresponds to the known toxicity profiles of both platinum agents (9–11). In the carbo-5FU cohort, 24.6% of the patients could not complete chemotherapy because of thrombocytopenia. If the platelet count was <100 × 10^9^/L on day 22 or day 43, the cycle was postponed. This usually resulted in omission of the third cycle because it could not be given within the radiotherapy interval.

Although OS was significantly better in the cisplatin cohort, the well-known prognostic factors were all advantageous for this cohort. After correcting for T stage, N stage, p16 status, and second primaries in multivariate analysis, we found that treatment regimen was not an independent prognostic factor for OS (12). Similar DFS, LRC, and distant metastasis–free interval were observed in both univariate and multivariate analysis.

Our results are similar to those of two previous comparative studies that demonstrated no significant difference in outcome between patients who received cisplatin and those who received carbo-5FU (13, 14). However, these single-center retrospective studies were restricted by statistical power and potential selection bias (13, 14).

Furthermore, we investigated whether treatment delay due to thrombocytopenia could have been caused by a relatively high carboplatin dose because dosing was based on body surface area rather than on creatinine clearance. However, we did not find an association between carboplatin AUC and grade 3 or grade 4 thrombocytopenia. Neither did we find a difference between cumulative carboplatin AUC of the first two cycles between patients who completed three cycles and those who completed two cycles.

Another interesting strategy that could reduce acute toxicity of concomitant CRT and further increase compliance comes from the Radiation Therapy Oncology Group 0129 trial. This study demonstrated similar efficacy of two cycles of high-dose cisplatin plus accelerated fractionation radiotherapy compared to three cycles of cisplatin plus standard fractionation (15). In the experimental arm, 87.8% of the patients completed two cycles of cisplatin whereas in the standard arm 69.0% completed three cycles. However, no difference in grade 3 or higher toxicity was found.

A limitation of our study is a difference in baseline characteristics between the cohorts due to the non-randomized retrospective design. Although this difference does not affect interpretation of chemotherapy completion rate, which was associated with treatment regimen, sex, and comorbidity, it does complicate interpretation of efficacy endpoints. However, a future randomized controlled trial investigating carbo-5FU vs. cisplatin is unlikely because the treatment focus has shifted toward immunotherapy and treatment de-escalation for low-risk p16-positive oropharyngeal cancer. Therefore, comparing patient cohorts from two tertiary care centers in the same country and geographic region with different institutional practice provides the best level of evidence attainable in current clinical practice. Another limitation of this study is that we were not able to retrieve reliable information on performance status, mucositis, skin toxicity, and alcohol consumption because this was not registered systematically.

To our knowledge, this is the first well-powered retrospective cohort study in which carbo-5FU and cisplatin as concomitant CRT for locally advanced HNSCC are compared with regard to chemotherapy completion. A lower chemotherapy completion rate was found for patients treated with carbo-5FU compared to patients treated with cisplatin. However, chemotherapy regimen was not independently associated with OS. We therefore believe that both chemotherapy regimens are viable treatment options for concomitant CRT in patients with locally advanced HNSCC.

## Data Availability Statement

The datasets generated for this study are available on request to the corresponding author.

## Author Contributions

SH, IK, JL, JB, and SO: analysis and interpretation of data. JG, BP, MVe, CL, JL, JV, JB, and SO: provision of study materials or patients. All authors are accountable for all aspects of the work, gave final approval of the manuscript, and contributed to drafting the manuscript or revising it critically.

## Conflict of Interest

BV has a consulting/advisory role for Philips Computational Pathology and is a scientific advisory board member for Visiopharm. JG has received research grants from Abbvie, Roche, and Siemens. MVe has a consulting/advisory role for Repare Therapeutics and has received travel/accommodation support from Repare Therapeutics. JB has a consulting/advisory role for MSD, Merck BV, and BMS, and has received travel/accommodation support from MSD. Furthermore, JB has participated in a speakers' bureau for BMS, and AstraZeneca. BP has a consulting/advisory role for Olympus Medical Systems and has received grants and travel/accommodation support from Olympus Medical Systems. JL has a consulting/advisory role for JBA. SO has received grants from Celldex. The remaining authors declare that the research was conducted in the absence of any commercial or financial relationships that could be construed as a potential conflict of interest.
