# Supplementary material for: Comparison of Carboplatin With 5-Fluorouracil vs. Cisplatin as Concomitant Chemoradiotherapy for Locally Advanced Head and Neck Squamous Cell Carcinoma
Source: Front Oncol. 2020 Jun 5;10:761. doi: 10.3389/fonc.2020.00761 (PMC7292049; doi:10.3389/fonc.2020.00761)
Supplement: Supplementary file 1 [file Data_Sheet_1.docx]

**Supplementary Figure 1.** Flowdiagram

Excluded (n = 116)

  No Squamous Cell Carcinoma (n = 26)

  No IMRT (n = 9)

  Not Oral Cavity, Oropharynx,

Hypopharynx or Larynx (n = 81)

Divided by Treatment Center

Assessed for eligibility (n = 652)

## Follow-Up

## Enrollment

UMCG (n=241)

 Received carbo-5FU (n=211)

 Did not receive allocated intervention (cetuximab) (n= 30)

## Allocation

AUMC (n = 295)

 Received 3-weekly cisplatin (n= 223 )

 Did not receive allocated intervention (weekly (cisplatin)(n= 45) or (cetuximab)(n= 27)

Discontinued intervention (n = 52)
Deaths (n = 61)
Lost to follow up (n=15)

Discontinued intervention (n = 83)
Deaths (n = 78)
Lost to follow up (n=5)

## Analysis

Analysed (n=223)

Analysed (n=211)

Abbreviations : UMCG; University Medical Center Groningen, AUMC; Amsterdam University Medical Center.
